# Development of insect resistant maize plants expressing a chitinase gene from the cotton leaf worm*, Spodoptera littoralis*

**DOI:** 10.1038/srep18067

**Published:** 2015-12-14

**Authors:** Gamal H. Osman, Shireen K. Assem, Rasha M. Alreedy, Doaa K. El-Ghareeb, Mahmoud A. Basry, Anshu Rastogi, Hazem M. Kalaji

**Affiliations:** 1 Biology Department, Faculty of Applied Sciences, Umm Al Qura University, Makkah- 21955, Kingdom of Saudi Arabia; 2 Agricultural Genetic Engineering Research Institute (AGERI), Agricultural Research Center, Giza, Egypt; 3 Biology Department, Faculty of Sciences, Taif University, Taif, Kingdom of Saudi Arabia; 4 Department of Cell Biochemistry, Faculty of Biochemistry Biophysics and Biotechnology, Jagiellonian University in Krakow, Gronostajowa 7, 30-387 Krakow, Poland; 5 Department of Plant Physiology, Faculty of Agriculture and Biology, Warsaw University of Life Science – SGGW, Ul. Nowoursynowska 159, 02-776 Warsaw, Poland

## Abstract

Due to the importance of chitinolytic enzymes for insect, nematode and fungal growth, they are receiving attention concerning their development as biopesticides or chemical defense proteins in transgenic plants and as microbial biocontrol agents. Targeting chitin associated with the extracellular matrices or cell wall by insect chitinases may be an effective approach for controlling pest insects and pathogenic fungi. The ability of chitinases to attack and digest chitin in the peritrophic matrix or exoskeleton raises the possibility to use them as insect control method. In this study, an insect chitinase cDNA from cotton leaf worm (*Spodoptera littoralis*) has been synthesized. Transgenic maize plant system was used to improve its tolerance against insects. Insect chitinase transcripts and proteins were expressed in transgenic maize plants. The functional integrity and expression of chitinase in progenies of the transgenic plants were confirmed by insect bioassays. The bioassays using transgenic corn plants against corn borer *(Sesamia cretica*) revealed that ~50% of the insects reared on transgenic corn plants died, suggesting that transgenic maize plants have enhanced resistance against *S. cretica*.

Production of agricultural crops are at risk due to the incidence of pests, especially weeds, pathogens and animal pests. Billions of dollars are spent globally each year for pest control and a large sum of money is lost due to inadequate control of pests. It is evident that the world food supply depends on effective protection of crops and animals from pests^1^,^2^,^3^,^4^. The chemical control of pests was efficacious and attractive during the forties and fifties of the last century. However, adverse effects of such chemicals quickly became apparent as evidenced by the accumulation of chemical pesticides in soil, water, air, agricultural products and in the body of animals. In addition, development of resistance in target organisms necessitated use of more selective and environmentally acceptable agents for pest control^5^,^6^. To fulfill the growing needs of human population and to prevent the environment, it is necessary to seek new, effective and environment-friendly ways of controlling pests. For instance, chitinases are being used to perturb structures containing chitin such as cuticle and peritrophic matrix (PM) that are essential for growth, development and survival of insects^7^,^8^. Cuticle is an extracellular matrix produced by most invertebrates, including insects. It is composed of chitin (a polymer of N-acetylgluosamine), a large assortment of proteins and lipids. Chitinases can help fungal and microbial pathogens to infect insect hosts by penetrating their cuticle. Additionally, chitinases affect the larval and adult PM that forms a chitinous tubular structure deposited by the gut epithelium^9^,^10^. Scanning electron microscopic examination has proved the ability of chitinases to perforate PM in midgut of cotton leaf worm larvae *in-vitro* at the concentration range of 0.1–10 μg/ml^11^.

All kinds of insects produce chitinases, which are essential for cuticle turnover and mobilization^12^. For instance, insects periodically shed their old exoskeleton and PM either continuously or periodically and resynthesize new ones^13^. Chitinases are among a group of proteins that insects use to digest the structural polysaccharide chitin in their exoskeletons and gut linings during the molting process^14^. Pesticidal activity is due to their ability to bind to chitin component of PM lining the insect gut and cuticle causing degradation of chitin containing matrices and death to the organisms^15^,^16^.

It is possible that the chitinase gene transfer technology will become as effective as the Bt gene transfer for the production of a pesticide free environment^17^. Alternatively, chitinase transgene may synergize the effect of Bt by decreasing the effective dose needed for insect control^18^,^19^. In fact, chitinase gene transfer technology may ultimately prove to be more important, since chitinase affects the growth and survival of both insect and fungal pathogens. It is generally assumed that plant chitinases, which belong to 18 glycosylhyrolases family, are effective against some insects when they are fed grain-based diets containing high concentrations of these enzymes^18^. Chitinase alone has been shown to significantly inhibit the insect feeding^20^. Crude chitinase preparations from *B. circulans* enhanced the toxicity of Bt kurstaki toward diamondback moth larvae^19^. Larvae of *C. fumiferana* died more rapidly when exposed to chitinase–Bt mixtures than when exposed to the enzyme or bacterium alone^20^,^21^. These studies have shown that insect chitinases, which belong to family 18 glycosylhydrolases, are good alternative for insect control.

Corn is considered the most important cereal crop after wheat and rice all over the world^22^. Corn borers (*S. cretica, Ostrinia nubilalis, Chilo agamemnon*) are serious insect pests in much of the corn growing areas around the world and responsible for significant loss of crop yield^23^. In addition, maize is susceptible to fungal diseases such as ear and stalk rot caused by *Fusarium moniliforme, Helminthosporium spp.,* and *Rhizoctonia zeae* and late welt caused by *Cephalosporium maydis*^24^. The development of a reliable transformation system could facilitate the introduction of useful genes conferring resistance to fungal diseases and insects into agronomical important cultivars. The addition of chitinolytic enzyme genes, whose encoded proteins adversely affect insect pests and fungal pathogens, to the repertoire of other defense genes in plants should enhance the effectiveness of this type of biotechnological control strategy. To date, only a few studies have been reported that directly used insect-derived chitinases as biopesticides for the control of pests. Elmenofy^6^ constructed a recombinant AcMNPV baculovirus expressing a group I chitinase from *M. sexta* under the control of the polyhedrin promoter. When the fourth instar larvae of *M. sexta* or *S. frugiperda* were injected with the recombinant virus, the chitinase was detectable in large amounts in the hemolymph. Liquefaction of infected *S. frugiperda* larvae occurred significantly earlier than when the insects were infected with a wild-type virus, indicating increased insecticidal activity^15^. A mixture of recombinant virus and purified recombinant protein were found to be more efficient in killing the ticks than recombinant virus and pure chitinase alone. Interestingly, even though purified recombinant chitinase has insecticidal effects. The objectives of this study were to isolate a chitinase gene from insects, to develop transgenic maize expressing chitinase and to test the transgenic lines against corn borer (*S. cretica*).

## Results

**Figure 1:**
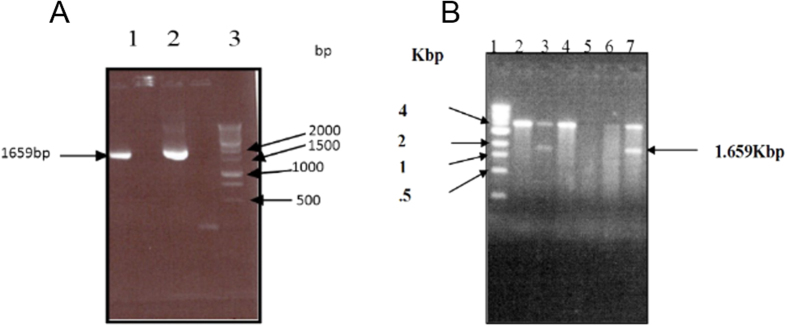
Chitinase cDNA cloning and isolation. Figure “A” represents the amplification of coding region of the chitinase gene of cotton leaf worm *S. littoralis* through RT-PCR and chitinase-specific primer pairs (lanes 1 and 2); lane 3 is the DNA size marker. The chitinase band was observed just above the 1500 bp marker band, which corresponds to a size of 1659 bp. Figure “B” represents screening for positive clones by digestion of recombinant PCRII plasmid DNA with *Eco R1*. Lane 1 indicates the 1 kb DNA maker. Lanes 3 and 7 are positive clones showing the insert DNA fragment for chitinase cDNA whereas, lanes 2, 4, 5 and 6 are plasmid clones without inserts.

**Figure 2:**
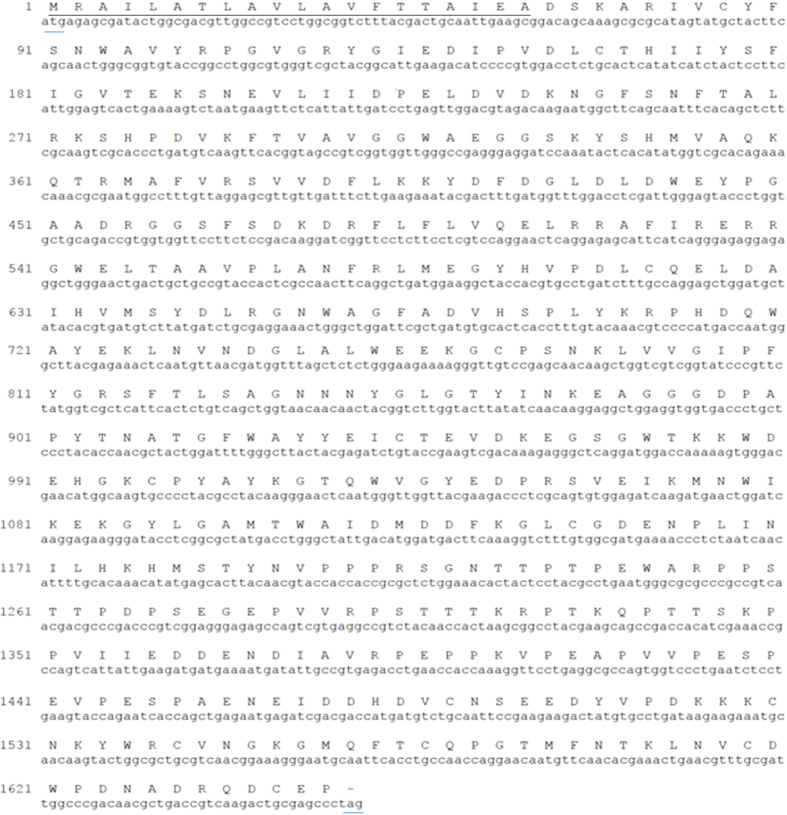
Nucleotide and deduced amino acid sequence of the *S. littoralis* chitinase gene. The open reading frame is from 1 to 1656 nucleotides, encoding a protein of 552 amino acids. The nucleotides are numbered on the left. Putative signal is underlined. The initiation codon and stop codon are underlined.

**Figure 3:**
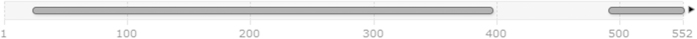
Schematic representation and putative signature domains of the *S. littoralis* (Accession # KP641331) chitinase. The figure indicates that the predicted protein has two different domains. The amino acid sequence between 21 to 396 represents the catalytic domain of the glycoside hydrolase gene (family 18) whereas the amino acid sequence between 490 to 552 represents the chitin binding domain. This analysis was accomplished using InterPro database.

**Figure 4:**
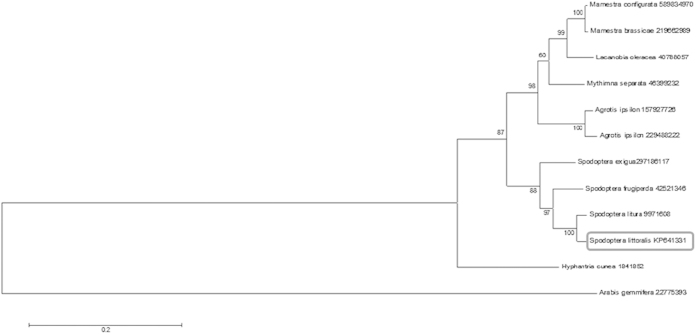
Chitinase phylogenetic analysis: The evolutionary history of *S. littoralis* (Accession # KP641331) chitinase gene (Square) was inferred by using the Maximum Likelihood method based on the Tamura-Nei model against the closely related known insect chitinases, with the chitinase of *Arabis gemmifera* (Accession # 22775393) used as an outlier. The tree with the highest log likelihood (–5711.5687) is shown. The percentage of trees in which the associated taxa clustered together is shown next to the branches (bootstrap value). There were a total of 1068 positions in the final dataset. Evolutionary analyses were conducted in MEGA6.

Synthesis and sequencing of a cDNA encoding an insect chitinase from cotton leaf worm: For cloning the insect chitinase cDNA, RT-PCR was performed using one pair of specific primers based on *Spodoptera frugiperda*^25^ chitinase gene (ChF1and ChR1) and total RNA isolated from the integuments of third instar larvae as described in the Materials and Methods section. PCR product was resolved on 1% agarose gel and a single band of expected size (~1.6 kbp) was observed as shown in Fig. 1A. The DNA fragment was eluted and cloned in pCRII vector using the Invitrogen kit. As shown in Fig. 1B, positive clone that contained the 1.6 kbp chitinase cDNA fragment (lanes 3 and 7) were identified. DNA sequencing was performed to determine the DNA sequence insert (by DNA sequencing facility at Kansas State University, USA) which confirmed the identity of this insert as the desired chitinase cDNA. The size of the coding region was found to be 1659 nucleotides, which encodes a protein comprised of 552 amino acids with an estimated molecular weight of 62.5 kDa Fig. 2. The protein structure was predicted by using “Phyre2” and the protein was found to have 16 helical segments and 18 β sheets that cluster into three groups. Group (I) and (III) form antiparallel β sheets while group (II) forms parallel β sheets as shown in Fig. 3. Signal peptide prediction shows that the predicted protein sequence has a signal peptide starting at amino acid 1 and ends at amino acid 20 with a predicted cleavage site between amino acids 20–21. Analysis of amino acids composition revealed that it contains 61 basic amino acids, 81 acidic, 169 hydrophobic amino acids, and 133 polar amino acids. A search of the nucleotide sequence databases for sequence similarity to cotton leaf worm chitinase revealed highest similarities with a chitinase from *Spodoptera litura*, with sequence identity of 98% (Fig. 4). Sequencing confirms the presence of chitinase gene in plasmid in right orientation and indicates the presence of active chitinase molecule.

## Verification of the Recombinant Plasmid Harboring the Insect Chitinase Gene

### Maize transformation and regeneration

**Figure 5:**
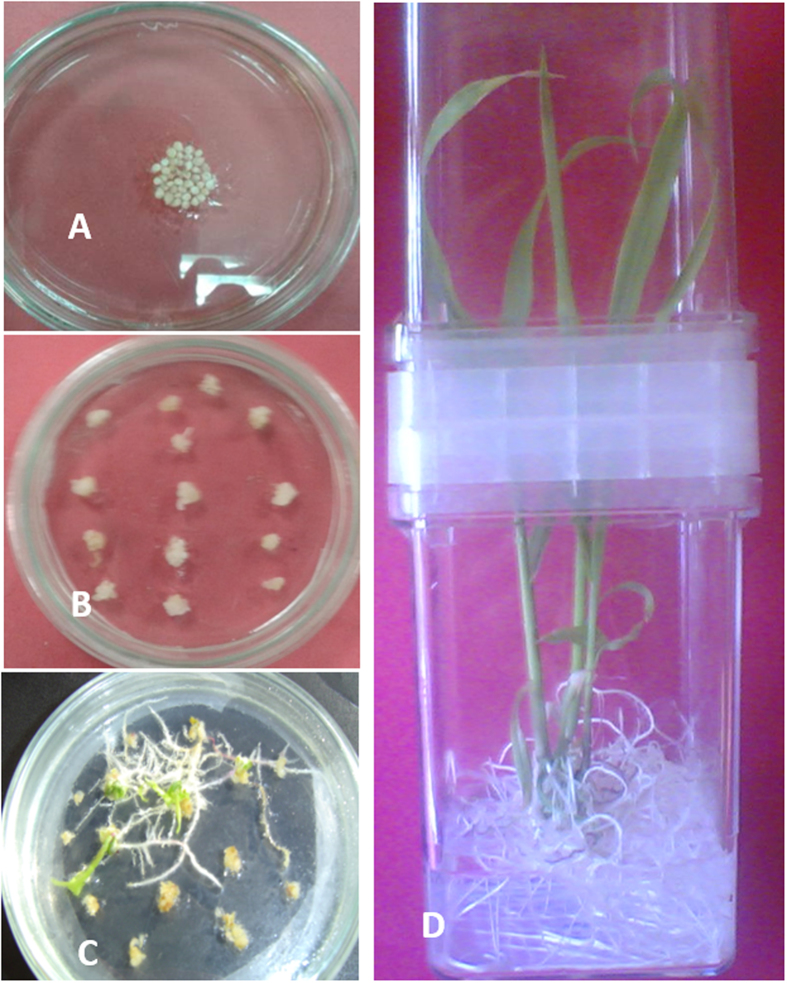
Maize transformation: **“A”** represents immature embryos derived calli prepared for bombardment by biolistic gene gun. “**B**” represents calli after bombardment (**C**,**D**) indicates maize shoots regenerated from putatively transformed calli.

The plasmid pChi-SB (harboring the insect chitinase and the *bar* genes. Immature maize embryos were bombarded once at 1100 psi, which yielded a number of regenerated putatively transgenic plantlets harboring the insect chitinase gene as well as the *bar* gene, as shown in Fig. 5. The results of the transformation experiments are summarized in Table 1. In the present study, we used the most regenerable maize inbred lines to express the introduced chitinase gene. The regenerated transgenic plants were subjected to molecular analysis as well as insect bioassays.

### Detection of the presence of the insect chitinase gene in putative transgenic plantlets

**Figure 6:**
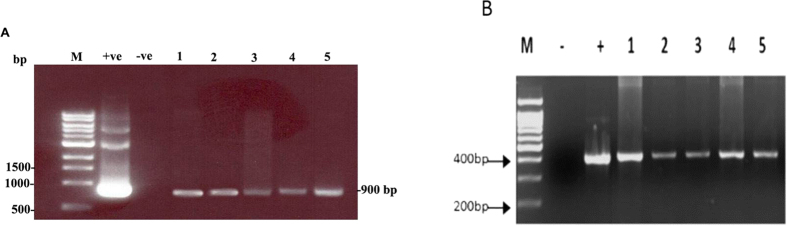
Presence of insect chitinase gene in transgenic maize: Figure represents the outcome of PCR reactions carried out for detection of transgenic chitinase DNA in genomic DNA samples isolated from putatively transgenic plants. (**A**). PCR products for plasmid pChi-SB, where ‘M’ is DNA marker 1kb; ‘ +ve’ is positive control using plasmid pChi-SB as template; ‘−ve’ is negative control using genomic DNA from an untransformed plant. Lanes 1–5 are genomic DNA samples from individual events of inbred resulting from biolistic gene gun transformation. (**B**). PCR product of partial-length bar gene. Lane ‘M’ is DNA marker; ‘ + ’ is positive control Chi-SB/Chitinase/bar plasmid, ‘−’ is negative control (non-transgenic line). Lane 1 to 5 are the inbreed lines of maize resulting from biolistic gene gun transformation.

PCR analysis was conducted to test the presence of the insect chitinase gene in the putatively transgenic maize plantlets. The amplification of the PCR product in the putative transgenic plants screened using appropriate sets of nested primer pairs to amplify 900 bp from the insect chitinase gene, confirmed the integration of the gene cassette into the plant genome (Fig. 6A). Moreover, PCR analysis carried out on the genomic DNA of putatively transgenic plantlets also revealed the presence of the 400 bp PCR fragment of the selectable marker *bar* gene, verifying transgenic events (Fig. 6B).

### Western blot analysis

**Figure 7:**
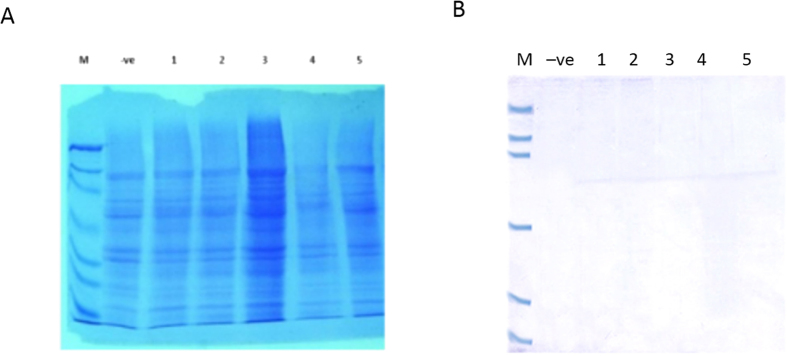
Insect Chitinase expression in transgenic plant: Figure represents the production of insect chitinase on transgenic maize plants. (**A**) Crude proteins separated on SDS-PAGE stained with Coomassie blue. ‘M’ is the protein marker lane; ‘−ve’ is negative control extract from untransformed maize and ‘1–5′ are protein extracts from transgenic maize. “B” represents the western blot of the respective samples shown in Coomassie stained gel in A probed with insect chitinase antibodies. (**M**) protein marker; (−ve) negative control extract from untransformed maize, (1–5) extracts of protein from individual events of inbred lines of maize resulting from biolistic gene gun transformation.

The crude protein extracts obtained from the regenerated putative transgenic maize plants were analyzed by SDS-PAGE, and also compared with non-transgenic maize (Fig. 7A). Western blot analysis of protein extracts of leaves from representative lines employing polyclonal antibodies raised against *M. sexta* chitinase showed the presence of a single prominent 62.5 kDa band corresponding to the expressed size, indicating that the transgene is being expressed constitutively (Fig. 7B). These experiments showed successful transformation of maize cultivars with insect chitinase expression.

### Insect toxicity Assay

**Figure 8:**
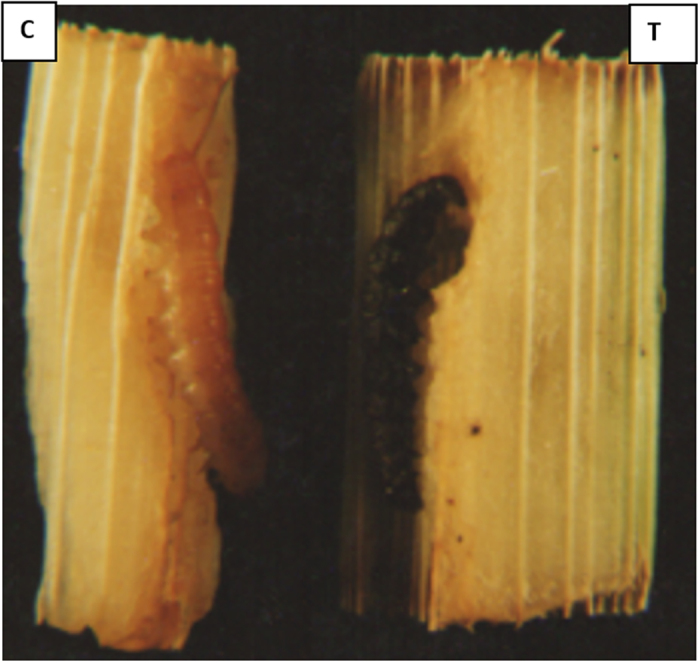
Effect of transgenic crops on insect morphology. Larvae of corn borer (*S. cretica*) turned black and got dead when reared on transgenic maize plants expressing insect chitinase (T) when compared with wild type maize plant (C).

Estimation of the toxicity of transgenic plants to insects was carried using a whole plant feeding assay. The first instar larvae of corn borer *(S. cretica)* were fed for 7 d in the laboratory, and a significant difference on insect mortality were observed. Five genotypes of transgenic maize plants were used in whole plant feeding assays. First instars larvae of corn borer *(S. ceritica)* allowed to feed on these plants had a mortality in the range of 63–70% (Table 2). The morphological differences in insect larvae is clearly visible when feed on transgenic maize plant (Fig. 8).

## Discussion

Chitinases are present in high concentrations in cereal grains known to be nontoxic to plants and higher vertebrates whereas they are toxic to plant pathogens such as insects and fungi. Transgenic plants expressing an insect chitinase gene have shown enhanced resistance to insect feeding in many studies. This is because of its capacity to degrade the linear polymer of chitin consisting of β-1,4-linked N-acetylglucosamines, which is an integral part of insect cuticle and PM. Hence transgenic crop overexpressing the insect chitinase are protected from the pathogenic fungi and pest insects. First studies that evaluated insect resistance of transgenic plants expressing an insect chitinase utilized transgenic tobacco plants and European corn borer^26^,^27^. In this study, the expression level of insect chitinase was found to be low, but even then the mortality rate of European corn borer was found significant when compared with the wild type. However no significant mortality was observed on *M. sexta* larva feeding on same transgenic tobacco line. The reason for this was attributed to the thickness of PM in case of *M. sexta* compared to European corn borer. In another study, transgenic papaya lines expressing the *M. sexta* chitinase gene showed significant tolerance to spider mites under field conditions^28^. On the other hand, transgenic potato plants expressing a chitinase from the coleopteran pest, *Phaedon cochleariae* revealed slightly positive effects on population growth of the aphid *M. persicae*^29^,^30^. This can be explained by the absence of the PM in aphids, which rendered the transgenic chitinase to be ineffective to the aphid population. This shows the limitation of chitinase based transgenic crop against the insects as an oral insecticide.

As the previous studies have demonstrated positive insecticidal effects of chitinase, it is important to include this strategy for generating the transgenic maize resistant to insects. Maize production is damaged by insects, due to which we need to improve resistance management strategies by developing transgenic lines of commonly cultivated maize varieties with resistance to most of the insects as well as fungi. Biological control of some soil born fungal diseases has been correlated with chitinase production. Bacteria-producing chitinases and or glucanases exhibit antagonism *in-vitro* against fungi [inhibition of fungal growth by plant chitinases and dissolution of fungal cell walls by a streptomycete chitinase and p-(1,3)-glucanase have been demonstrated]. The importance of chitinase activity was demonstrated by the loss of biocontrol efficacy in *Sewatia marcescens* mutants in which the chiA gene had been inactivated^31^. Molecular techniques have also facilitated the introduction of beneficial traits into rhizosphere competent and model organisms to produce potential biocontrol agents. A recombinant *Escherichia coli* expressing the chiA gene from *S. marcescens* was effective in reducing disease incidence caused by *Sclerotium rolfsii* and *Rhizoctonia solani*^32^. In other studies, chitinase genes from *S. marcescens* have been expressed in *Pseudomonas* sp. and the plant symbiont *Rhizobium meliloti*. The modified *Pseudomonas* strain was shown to control the pathogens *E oxysporum f. sp. redolens* and *Gauemannomyces graminis var. tritici*^33^. Numerous plant chitinase genes or cDNAs have been cloned. In a successful case, transgenic tobacco plants were generated which constitutively expressed a bean endochitinase gene under the control of the cauliflower mosaic virus 35s promoter. The transgenic tobacco plants were less susceptible to infection by *Rhizoctonia solani*, and either the disease development was delayed or they were not affected at all^34^. In conclusion, we have generated transgenic maize plant that overexpresses an insect chitinase. The chitinase cDNA from *S. littoralis* was isolated and transferred in different genotype of maize plant widely grown in Egypt. The expression of transgenic insect chitinase was observed and found to be overexpressed in regenerated transgenic maize. The insect resistance was also found to be significantly improved in the case of transgenic maize plant. This study is the first attempt to improve the maize productivity in Egyptian maize varieties so that farmers will get maximum benefits by protecting the crops in the field as well as during storage of grains.

## Materials and Methods

Insect samples of *S. littoralis* and *S. cretica* were obtained from the insectary at the Agricultural Genetic Engineering Research Institute (AGERI), ARC-Egypt.

### Synthesis and cloning of cDNA encoding an insect chitinase gene

Total RNA was isolated from the integuments of third instar larvae of *S. littoralis* using the QIAGEN kit for total RNA isolation. First strand cDNA was synthesized from the total RNA isolated using cDNA reverse transcriptase kit from Invitrogen. The total cDNA obtained was then used as template for amplification of chitinase cDNA, using insect chitinase-specific oligonucleotide primers (Ch1F- 5′ATGAGAGCGATACTGGCG3′ and Ch1R-5′ CTAGGGCACGCAGTCTTG 3′), specific to a *S. frugiperda*^25^ chitinase gene. Insect chitinase cDNA amplified this way was cloned in PCRII cloning vector (Invitrogen). Polymerase chain reaction was performed in a 50 μl reaction volume. A mixture containing 10 mM Tris-HCl (pH 8.3), 50 mM KCl, 2 mM MgCl2, 0.01% (W/V) gelatin, 200 μM each of dGTP, dATP, dCTP and TTP, 2.5 units of RTS Taq DNA polymerase, 0.2 μM of the above primers for the synthesis of cDNA. The amplification was carried out for 35 cycles, each consisting of a denaturing step at 94 °C for 30 sec, annealing step at 55 °C for 30 sec, and extension at 72 °C for 2 min and the last cycle had a 7 min extension at 72 °C. The amplified products were separated on a 1% agarose gel.

### DNA manipulation and nucleotide sequence

Recombinant plasmid DNA isolation was carried out essentially as described by Sambrook and Russell^35^ using QIAGEN spin columns. Sequence determination was carried out by the dideoxy chain termination method^36^ using the PRISM sequence fluorescent dye-labeled dideoxy nucleotide kit (PE Applied Biosystem inc.). The complete nucleotide sequence of the ~1600 nucleotides-long coding region of chitinase cDNA was obtained using M13 forward 5′CTGGCCGTCGTTTTAC3′ and reverse 5′GTCGTGACTGGGAAAAC3′ primers in addition to one nested primer 5′ACTGACTGCTGCCGTACCACT3′ (549–569).

### Construction of plant expression vectors

**Figure 9:**
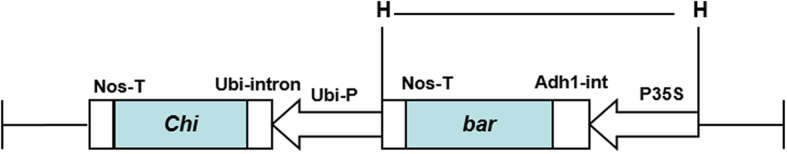
Expression vector: Schematic diagram of the expression vector pChi-SB for maize transformation.

The plasmid pChi-SB containing the *bar* gene as a selectable marker and insect chitinase was constructed Fig. 9. The bar gene was cut from the plasmid pAB8 (4799 bp) with *HindIII* and inserted in the *HindIII* site of pAHC25. The insect chitinase gene was also inserted in the plasmid pAHC25 by using *EcoRI*. The large scale preparation of constructed pChi-SB has been done by using the Wizard maxi prep kit (Promega) for plasmid isolation. The plasmid DNA was diluted to a final concentration of 1μg/μl and used for plant transformation.

### Plant materials and culture initiation

Seeds from the desired maize genotypes were sown in the field at different intervals and were used as a continuous source of immature embryos as explants. A number of embryogenic maize genotypes have been selected for transformation experiments (Gz639, Gz624, Gz649, Gz650 and A188). Immature zygotic embryos of different maize genotypes were used as target explants. Maize ears were harvested from the field-grown plants, 10 to 15 days post pollination. Ears were surface sterilized (by treating with 5.25% hypochlorite with 0.1% TWEEN 20) and then washed three times with sterile distilled water. Immature embryos (1–1.5 mm in length) were aseptically excised as described previously^37^ and incubated for 4 to 7 days at 25 °C on N6-based callus induction medium (N6-Ag) containing 1.7 mg/l silver nitrate and 2% sucrose^38^,^39^.

### Particle bombardment and selection

The gene gun (Bio-Rad, Biolistic PDS-1000/He) was used for the transformation of maize explants. Several experiments were carried out on maize immature embryos from all lines. Four hours before bombardment, embryos were placed in the center of the plates containing osmotic medium (as described in El-Itriby *et al.*^40^). The osmotic treatment was continued for 16 hours after bombardment. Embryogenic scutellar tissues were bombarded once at 1100 psi with sterilized gold particles coated with plasmid DNA according to the modified protocol described in Zhong *et al.*^41^. Transformed maize tissues were incubated in darkness at 25 °C for four days. Selection process was carried out by transferring the bombarded embryos to N6-Ag medium containing 2 mg/l bialaphos for four weeks for selection of calli expressing the *bar* gene selectable marker with one subculture. Putatively transformed calli were further transferred to N6-Ag medium containing 3 mg/l bialaphos and incubated under the same conditions for four weeks with one subculture.

### Maize regeneration

After four rounds of selection, bialaphos-resistant calli were regenerated as described in El-Itriby *et al.*^40^ by transferring them to regeneration medium, RM1, followed by RM2 (containing 3 mg/l bialaphos) and were incubated under fluorescent light (250 μmol m^–2^ s^–1^). The regenerated shoots were rooted on RM3 medium containing 3 mg/l bialaphos. Putatively transgenic plantlets were acclimatized in the biocontainment green house, at 28 °C with a 16 h photoperiod. Healthy rooted plantlets were transferred to pots containing a mixture of peat moss: soil (1:1).

### Evaluation of the Putative Transgenic Plants

#### Analysis of the insertion of vector sequences into the maize genome

Total genomic DNA from the shoot of putative transgenic and untransformed plants (control) was isolated using DNeasy kit (QIAGEN). PCR analysis was conducted to detect the presence of the insect chitinase gene in genomic DNA of putative transgenic and control plants using two specific forward and reverse nested primers: F 5′T G G C T T C A G C A A T T T C A C A G3′, R 5′C T C C T G A G T T C C T G G A C G A G3′. After denaturation for five minutes at 94 °C, PCR reactions were carried out for 35 cycles using the following temperature sequence: 94 °C for 1 min, 55 °C for 30 sec and 72 °C for 1 min. In addition, PCR was also conducted to confirm the presence of the *bar* gene in genomic DNA from the putative transgenic plants using the specific forward and reverse primers for the *bar* gene: bar F 5′TACATCGAGACAAGCACGGTCAACT3′ and bar R 5′ACGTCATGCCAGTTCCCGTG3′. Products were size-separated on a 1% agarose gel containing ethidium bromide and observed under UV light.

#### Western blotting

To extract proteins, 0.5 g of mature healthy shoots were ground in liquid nitrogen with 0.2 M Tris-HCl buffer pH 8, 2% w/v SDS, 10% sucrose and 1% 2- mercaptoethanol (for detailed protocol see Laemmli^42^). Proteins were separated by SDS PAGE using 12% gels. Western blot analysis of proteins using an antibody to an insect chitinase from *Manduca sexta* allowed immunological confirmation of the identity of the protein produced in cotton leaf worm. The intensity of the stained band by western blot analysis depends on the total amount of protein in the band. The method used was the modified protocol described previously in Towbin *et al.*^43^ and Burnette^44^ Western blotting was performed using a rabbit polyclonal antibody raised against chitinase 535 from *Manduca sexta*. The antibody was kindly provided by Dr. Muthukrishnan, Department of Biochemistry at Kansas State University, USA. The protein extract was run on a gel containing 12% polyacrylamide, and then transferred to a polyvinylidenedifluoride (PVDF) membrane using a semi-dry blotting unit (Bio- Rad) overnight at 40 V. After protein blotting, the membranes were blocked by soaking in milk–based blocking buffer (5% powdered milk in 0.5% Tween 20) for 2 h at room temperature, then probed with the antiChi-535 antibody (1:1000 dilution in blocking buffer) as a primary antiserum for 4 h at room temperature. The membrane was washed three times with TBS (50 ml/wash for 15 min) to remove excess antibodies, and then the immune-reactive protein bands were visualized using alkaline phosphates–conjugated anti-rabbit (IgG-AP) as a secondary antibody (1:10,000 dilution in blocking buffer) for 1 h. The membrane was washed three times with TBS (50 ml/wash for 15 min) without sodium azide to remove excess antibodies and then with PBS three times (50 ml/wash for 15 min). The alkaline phosphatase activity was determined by incubating the membrane with an equal volume of Nitro Blue Tetrazolium (NBT) and 5-bromo-4-chloro-3-indolyl phosphate (BCIP) at room temperature for 10 min.

#### Insect bioassay

All positive transgenic plants expressing the chitinases protein were tested for lethality of the lepidopteron corn borer (*S. cretica*) larvae using a whole plant-feeding assay. The test plants were individually infested with 3 replicates of 10 each of 1st instar larvae of *S. cretica* for 7 d at 25–30 °C under 8/16 h light/dark regime. Bioassays were repeated three times. Mortality was scored daily until death or pupation. The control treatments utilized non-transgenic whole plants.

## Additional Information

**How to cite this article**: Osman, G.H. *et al.* Development of insect resistant maize plants expressing a chitinase gene from the cotton leaf worm, *Spodoptera littoralis*. *Sci. Rep.* **5**, 18067; doi: 10.1038/srep18067 (2015).

## Figures and Tables

**Table 1:** Summary of transformation experiments of five maize genotypes using the biolistic gene gun.

Exp. No.	Maize Genotype	Source of immature embryos	Number of bombarded immature embryos	Number of regenerated transgenic plants shoots regenerated
1	A188	Field	211	11^ab^
2	Gz639	Field	280	12^a^
3	Gz649	Field	400	10^bc^
4	Gz624	Field	80	8^d^
5	Gz650	Field	80	9.0^cd^

Numbers with the same letters are not significantly different at 5%.

**Table 2:** Toxicity of transgenic plant to larvae of corn borer (*S. cretica*).

Exp. number	Maize Genotype	NUMBER OF INSECTS	Dead insects	MORTALIY %
1	**A188**	**30**	19 ± 1.1	63.33 ± 3.67
2	**Gz639**	**30**	20 ± 2	66.67 ± 6.67
3	**Gz649**	**30**	18 ± 0.3	60 ± 1
4	**Gz624**	**30**	21 ± 3.5	70 ± 10.5
5	**Gz650**	**30**	19 ± 1.25	63.33 ± 4.17
6	**Control**	**30**	4 ± 3	13.33 ± 10

Numbers with the same letters are not significantly different at 5%.
