# Erratum: Development of insect resistant maize plants expressing a chitinase gene from the cotton leaf worm*, Spodoptera littoralis*

**DOI:** 10.1038/srep20449

**Published:** 2016-02-25

**Authors:** Gamal H. Osman, Shireen K. Assem, Rasha M. Alreedy, Doaa K. El-Ghareeb, Mahmoud A. Basry, Anshu Rastogi, Hazem M. Kalaji

Scientific Reports 5: Article number: 1806710.1038/srep18067; published online: 12142015 updated: 02252016.

In this Article, Shireen K. Assem is incorrectly listed as being affiliated with ‘Biology Department, Faculty of Applied Sciences, Umm Al Qura University, Makkah- 21955, Kingdom of Saudi Arabia’. The correct affiliation is listed below:

Agricultural Genetic Engineering Research Institute (AGERI), Agricultural Research Center, Giza, Egypt

